# Author Correction: The effect of inhibition of receptor tyrosine kinase AXL on DNA damage response in ovarian cancer

**DOI:** 10.1038/s42003-023-05139-9

**Published:** 2023-07-20

**Authors:** Xun Hui Yeo, Vignesh Sundararajan, Zhengwei Wu, Zi Jin Cheryl Phua, Yin Ying Ho, Kai Lay Esther Peh, Yi-Chia Chiu, Tuan Zea Tan, Dennis Kappei, Ying Swan Ho, David Shao Peng Tan, Wai Leong Tam, Ruby Yun-Ju Huang

**Affiliations:** 1 Genome Institute of Singapore (GIS), Agency for Science, Technology and Research (A*STAR), 60 Biopolis Street, Genome, Singapore, 138672 Republic of Singapore; 2 Cancer Science Institute of Singapore, National University of Singapore, 14 Medical Drive, Singapore, 117599 Republic of Singapore; 3 Bioprocessing Technology Institute (BTI), Agency for Science, Technology and Research (A*STAR), 20 Biopolis Way, Centros, Singapore, 138668 Republic of Singapore; 4 Graduate Institute of Oncology, College of Medicine, National Taiwan University, Taipei, Taiwan; 5 Department of Biochemistry, Yong Loo Lin School of Medicine, National University of Singapore, 10 Medical Drive, Singapore, 117597 Republic of Singapore; 6 NUS Center for Cancer Research, Yong Loo Lin School of Medicine, National University of Singapore, Singapore, Republic of Singapore; 7 Department of Haematology-Oncology, National University Cancer Institute, Singapore, Republic of Singapore; 8 School of Biological Sciences, Nanyang Technological University, 60 Nanyang Drive, Singapore, 637551 Republic of Singapore; 9 School of Medicine, College of Medicine, National Taiwan University, Taipei, Taiwan; 10 Department of Obstetrics & Gynaecology, Yong Loo Lin School of Medicine, National University of Singapore, Singapore, Republic of Singapore

**Keywords:** Ovarian cancer, Targeted therapies

Correction to: *Communications Biology* 10.1038/s42003-023-05045-0, published online 22 June 2023.

In the original version of the Article, Figure 5 contained misaligned labels and Figure 6 had erroneous quantification values, and the figure legends to Figure 2 and 6 contained omissions and mis-labelling.

Previous Figure 5
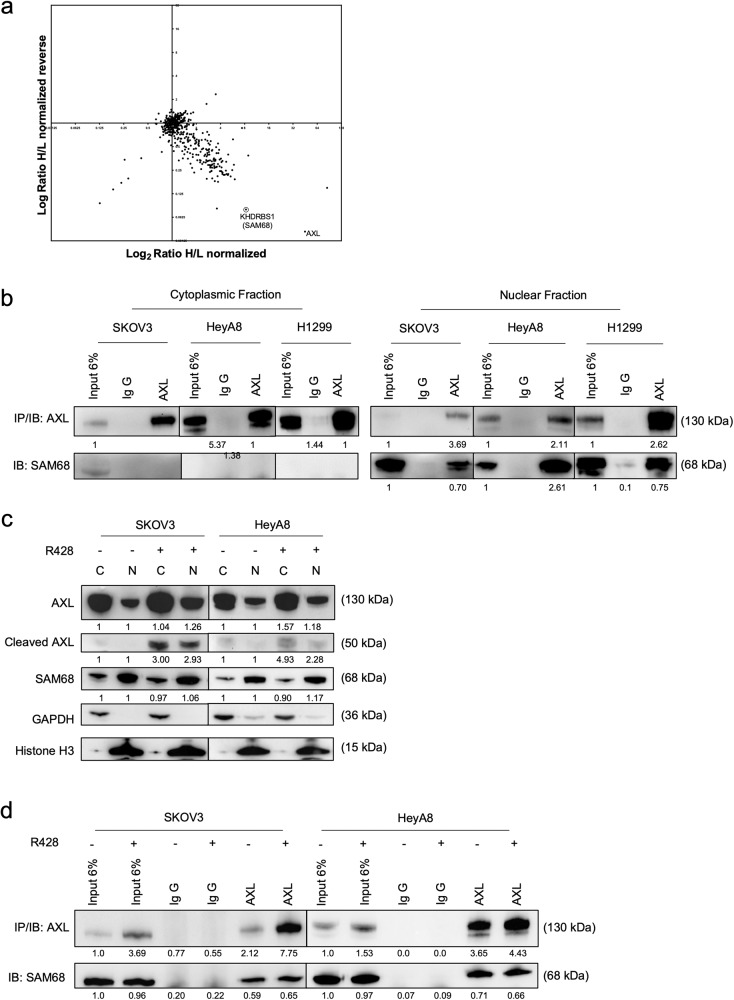


Corrected Figure 5
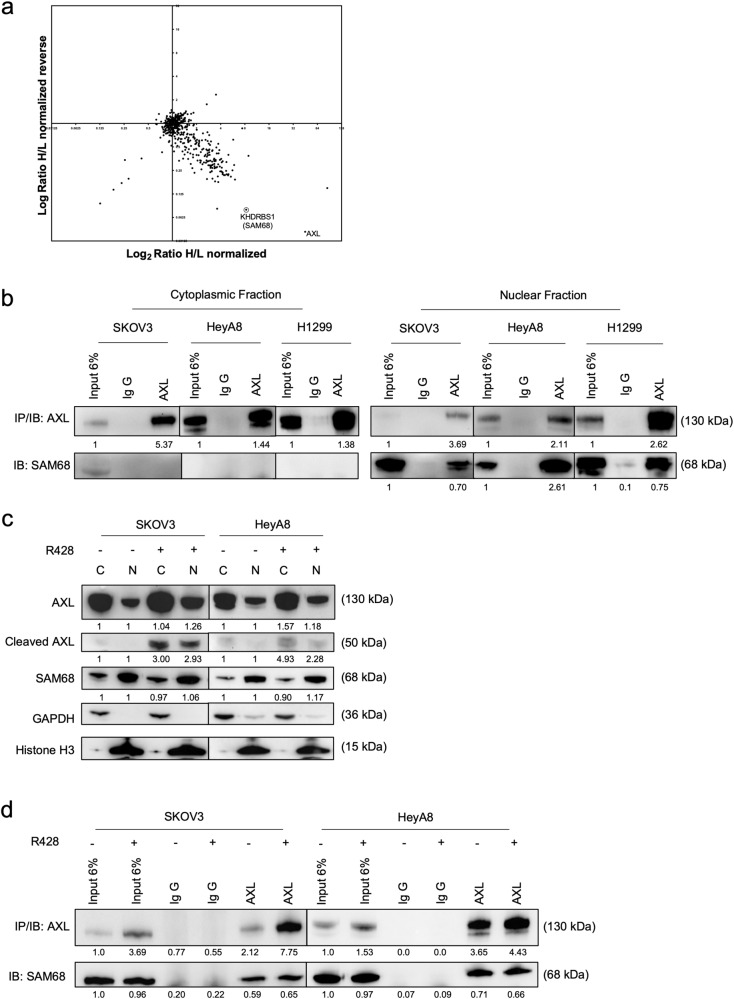


Previous Figure 6
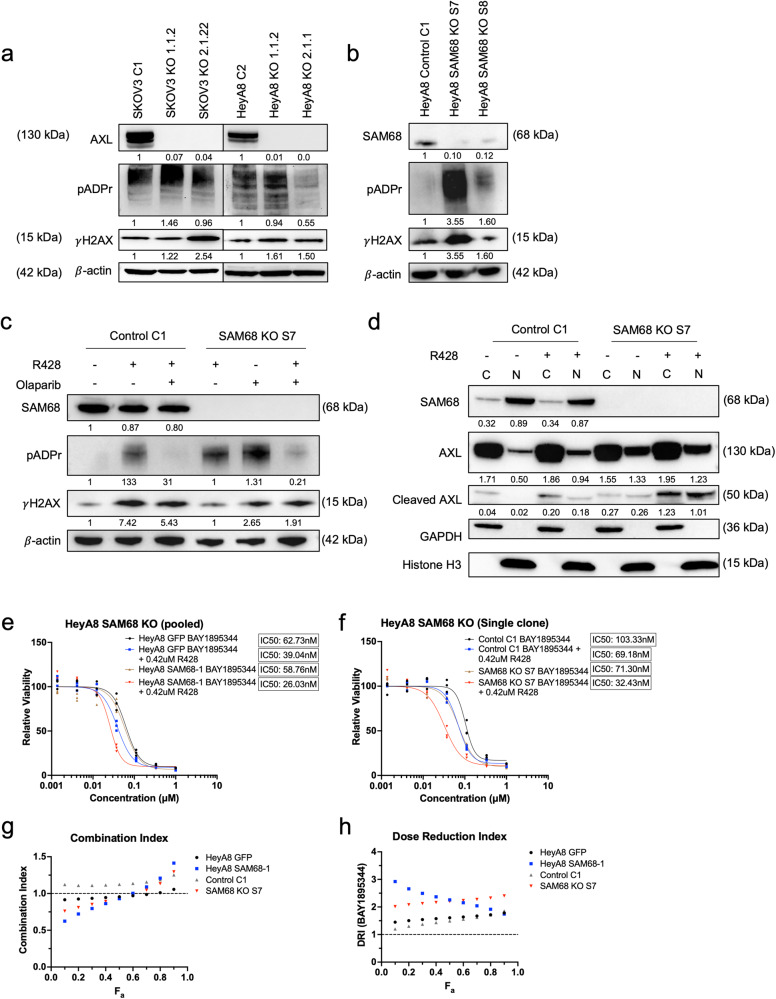


Corrected Figure 6
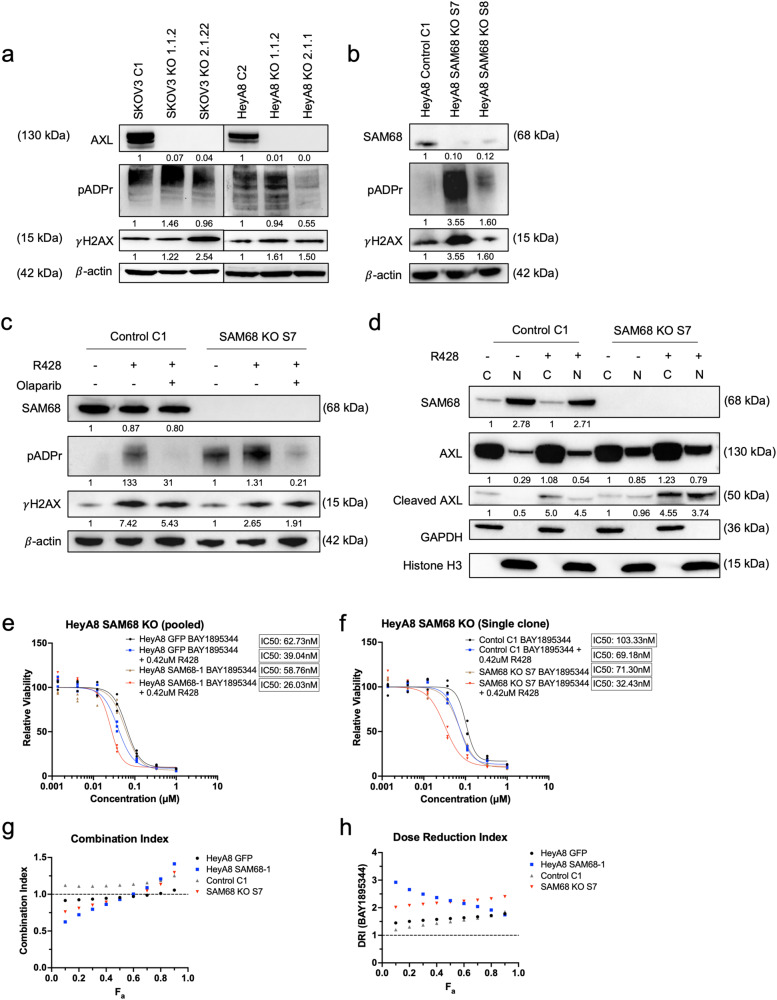


This has been corrected in the PDF and HTML versions.

