# Supplementary material for: The effect of inhibition of receptor tyrosine kinase AXL on DNA damage response in ovarian cancer
Source: Commun Biol. 2023 Jun 22;6:660. doi: 10.1038/s42003-023-05045-0 (PMC10287694; doi:10.1038/s42003-023-05045-0)
Supplement: Supplementary file 2 — Supplementary Information [file 42003_2023_5045_MOESM2_ESM.pdf]

Supplementary Figures

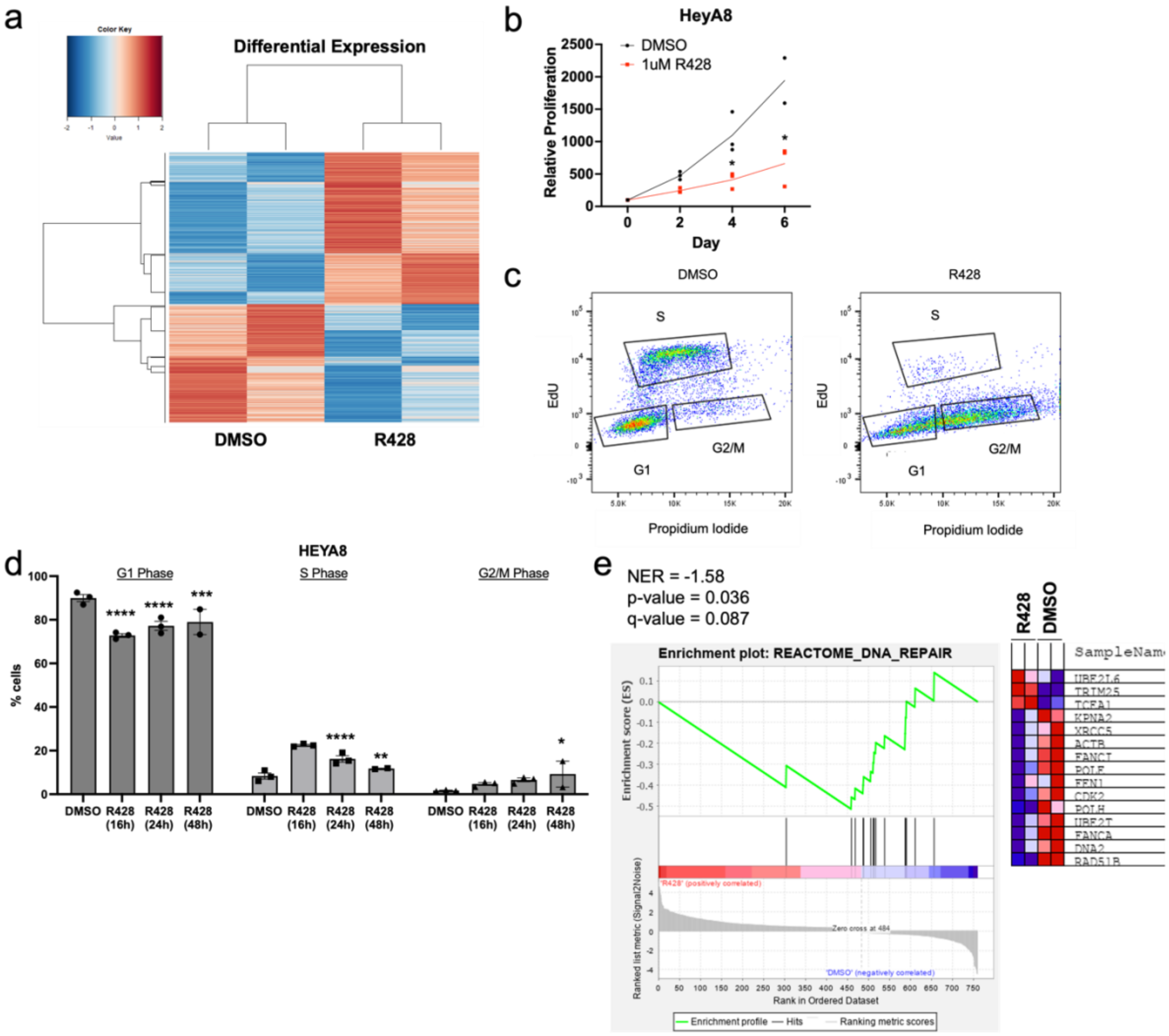

Supplementary figure 1: AXL inhibition associated with downregulation of cell cycle, mitosis and DNA replication

(a) Heat map of differentially regulated genes upon 24 hours R428. Data represent log_2 fold change </> 0.5, *p*-value < 0.05 (b) Proliferation curve of HeyA8 treated with R428 compared to DMSO (c) Representative flow cytometry plot of cell cycle analysis in HeyA8 treated with R428 for 24 hrs compared to DMSO. 10,000 events were recorded for each sample (d) Quantification of cell cycle analysis of HeyA8 treated with R428 for 16 hrs and 24 hrs showing the percentage of cells in different phases of cell cycle (G_1, S, G_2/M [G_2 and mitosis]). Data represent mean ± s.e.m.; $*p < 0.05$, $**p < 0.01$, $***p < 0.001$, $****p < 0.0001$, determined by Dunnett's multiple comparisons test (e) GSEA analysis showing negative enrichment of mitotic cell cycle and DNA repair pathways upon AXL inhibition

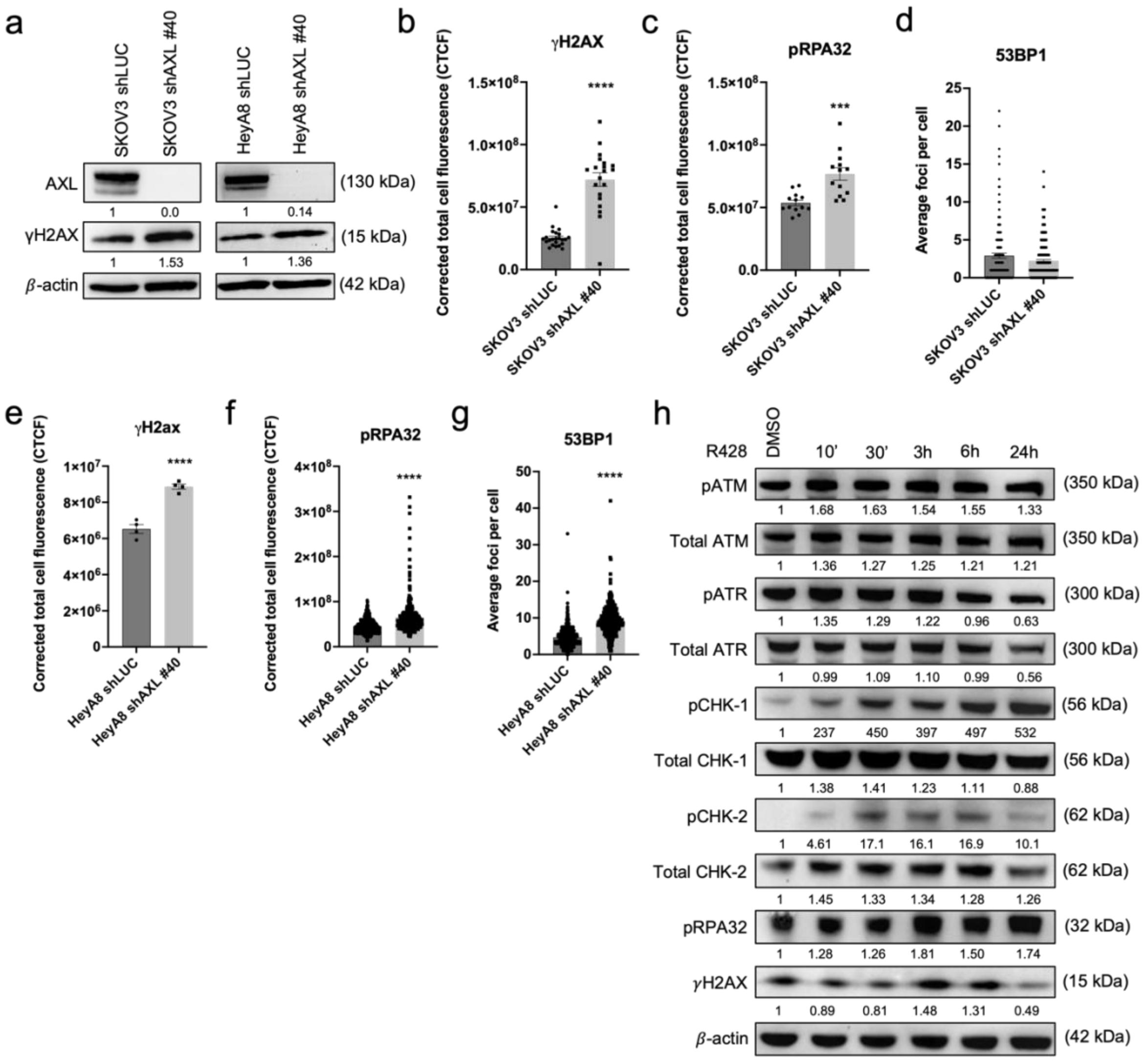

Supplementary figure 2: AXL inhibition increases DNA damage and DNA damage response pathways

(a) AXL knockdown in SKOV3 and HeyA8 (b) Fluorescence intensity quantification of γH2AX in SKOV3 shLUC Vs shAXL #40 (c) Fluorescence intensity quantification of pRPA32 in SKOV3 shLUC Vs shAXL #40 (d) Foci quantification of 53BP1 in SKOV3 shLUC Vs shAXL #40 (e) Fluorescence intensity quantification of γH2AX in HeyA8 shLUC Vs shAXL #40 (f) Fluorescence intensity quantification of pRPA32 in HeyA8 shLUC Vs shAXL #40 (g) Foci quantification of 53BP1 in HeyA8 shLUC Vs shAXL #40. Data in (c – g) represent mean ± s.e.m.; *$p < 0.05$, **$p < 0.01$, ***$p < 0.001$, determined by two-tailed t-test with statistical significance with Welch's correction (h) Immunoblot of DDR markers upon AXL inhibition at the indicated time points in HeyA8. Numbers below blots reflect protein band intensity normalised against DMSO

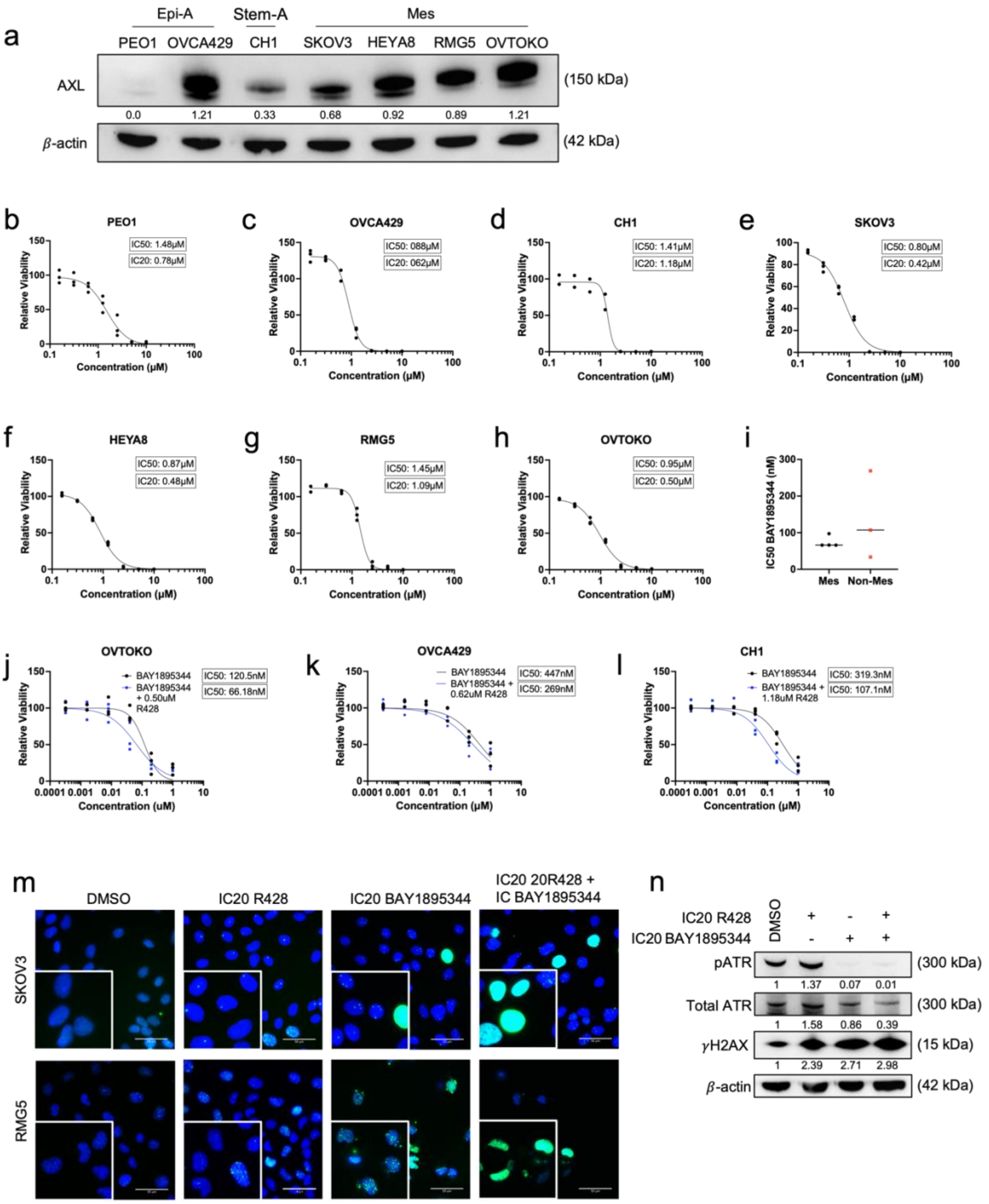
a
Epi-A
Stem-A
Mes
PEO1
OVCA429
CH1
SKOV3
HEYA8
RMG5
OVTOKO
AXL
(150 kDa)
0.0
1.21
0.33
0.68
0.92
0.89
1.21
β-actin
(42 kDa)
b PEO1 IC50: 1.48μM IC20: 0.78μM
c OVCA429 IC50: 088μM IC20: 062μM
d CH1 IC50: 1.41μM IC20: 1.18μM
e SKOV3 IC50: 0.80μM IC20: 0.42μM
f HEYA8 IC50: 0.87μM IC20: 0.48μM
g RMG5 IC50: 1.45μM IC20: 1.09μM
h OVTOKO IC50: 0.95μM IC20: 0.50μM
Relative Viability
Concentration (μM)
i IC50 BAY1895344 (nM)
Mes
Non-Mes
j OVTOKO
BAY1895344
BAY1895344 + 0.50uM R428
IC50: 120.5nM
IC50: 66.18nM
k OVCA429
BAY1895344
BAY1895344 + 0.62uM R428
IC50: 447nM
IC50: 269nM
l CH1
BAY1895344
BAY1895344 + 1.18uM R428
IC50: 319.3nM
IC50: 107.1nM
m
DMSO
IC20 R428
IC20 BAY1895344
IC20 20R428 + IC BAY1895344
SKOV3
RMG5
n
IC20 R428
IC20 BAY1895344
DMSO
pATR
(300 kDa)
1 1.37 0.07 0.01
Total ATR
(300 kDa)
1 1.58 0.86 0.39
γH2AX
(15 kDa)
1 2.39 2.71 2.98
β-actin
(42 kDa)

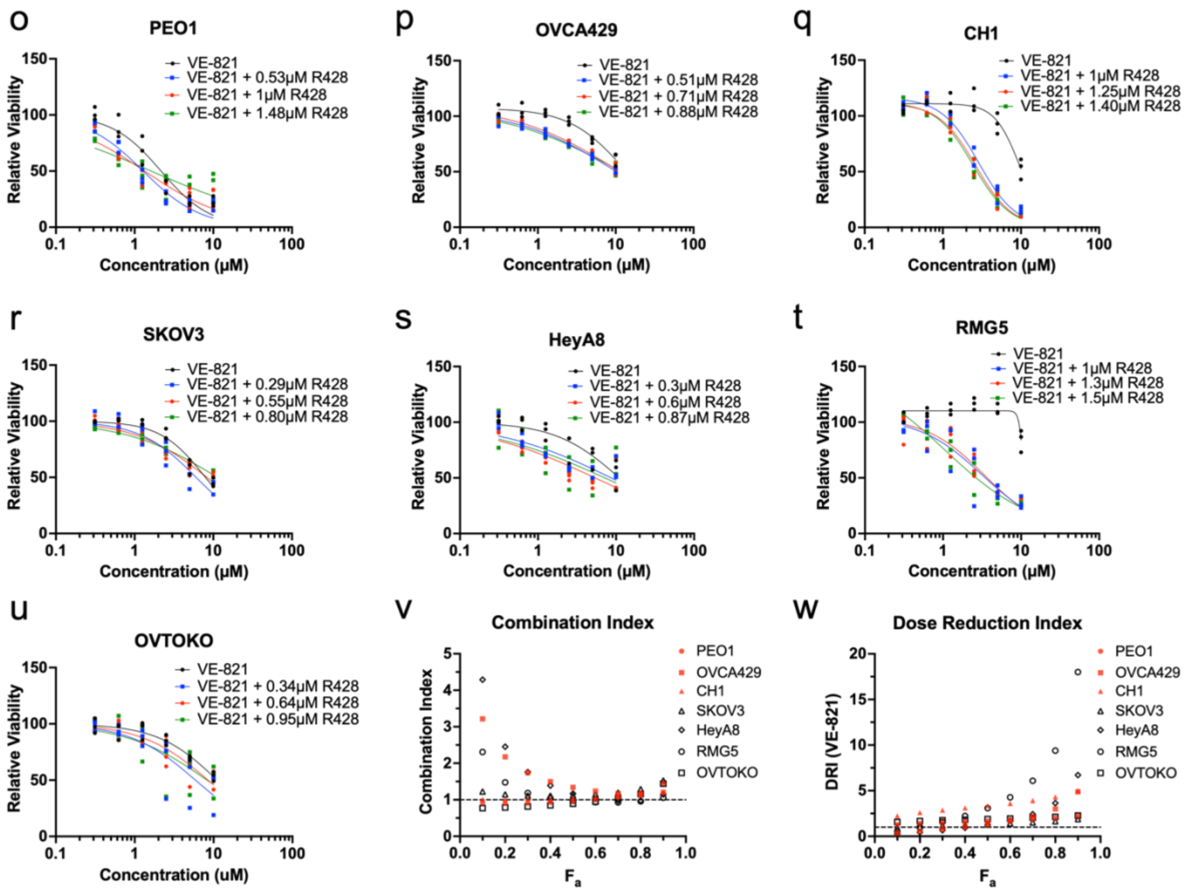

Supplementary figure 3: AXL inhibition sensitises cells to ATR inhibition

(a) Immunoblot of AXL expression in a panel of ovarian cancer cell lines. Dose response curves of R428 with respective IC_{50} and IC_{20} in (b) PEO1 (c) OVCA429 (d) CH1 (e) SKOV3 (f) HeyA8 (g) RMG5 (h) OVTOKO. (i) IC_{50} values of BAY1895344 in OC cell lines after sensitization with R428. Combination treatment of R428 with BAY1895344 in (j) OVTOKO (k) OVCA429 (l) CH1. (m) Immunofluorescence staining of γH2AX in SKOV3 and RMG5 cell lines treated with combination treatment of AXL and ATR inhibition. Blue, DAPI; Green, γH2AX. (n) Immunoblot of DDR markers in SKOV3 treated with combination treatment of AXL and ATR inhibition. Numbers below blots reflect protein band intensity normalised against DMSO. Combination treatment of $IC_{10}/IC_{30}/IC_{50}$ R428 with VE-821 in (o) PEO1 (p) OVCA429 (q) CH1 (r) SKOV3 (s) HeyA8 (t) RMG5 (u) OVTOKO (v) Combination index of R428 and VE-821 (w) Dose reduction index of VE-821. F_a: Fraction affected

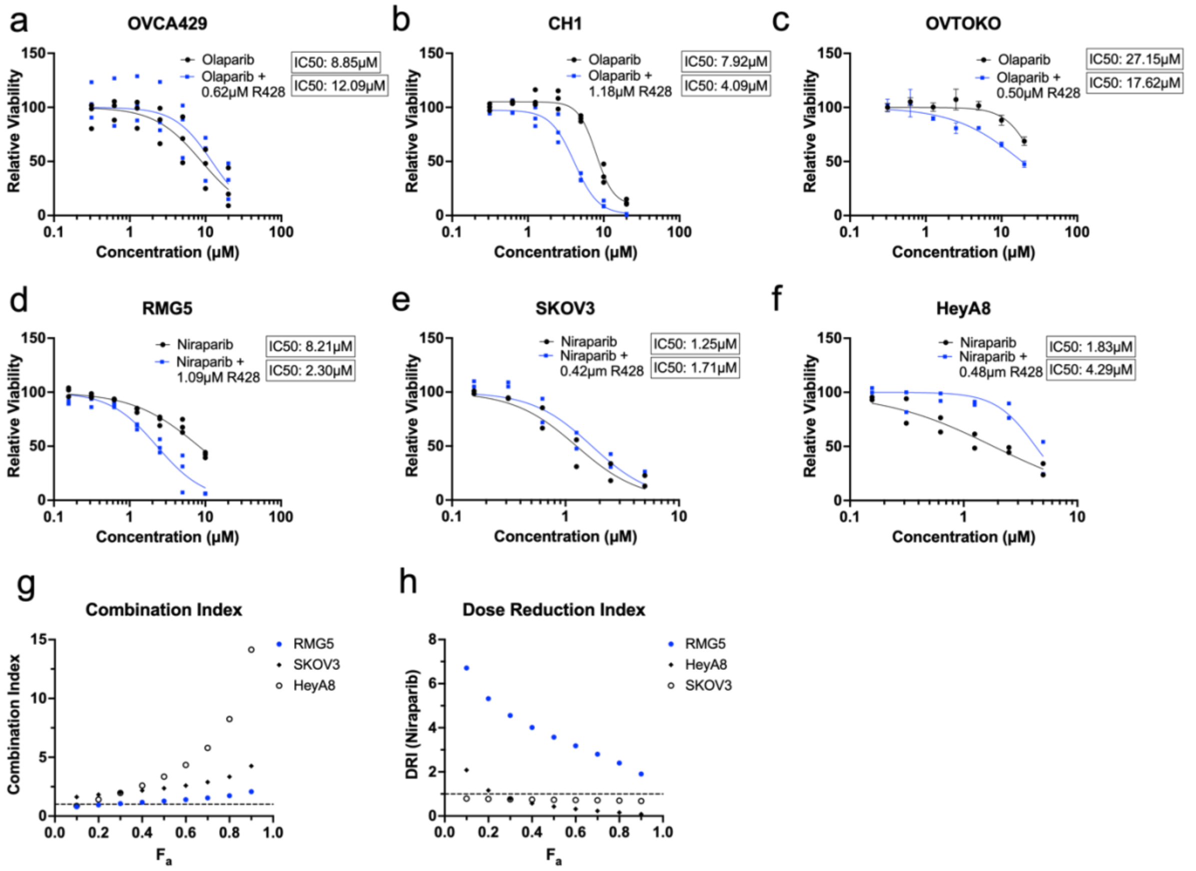

Supplementary figure 4: AXL inhibition sensitises *BRCA* mutant cells to PARP1 inhibition

Combination treatment of R428 with Olaparib in (a) OVCA429 (b) CH1 (c) OVTOKO. Combination treatment of R428 with Niraparib in (d) RMG (e) SKOV3 (f) HeyA8 (e) Combination index of R428 and Niraparib (f) Dose reduction index of Niraparib. F_a: Fraction affected

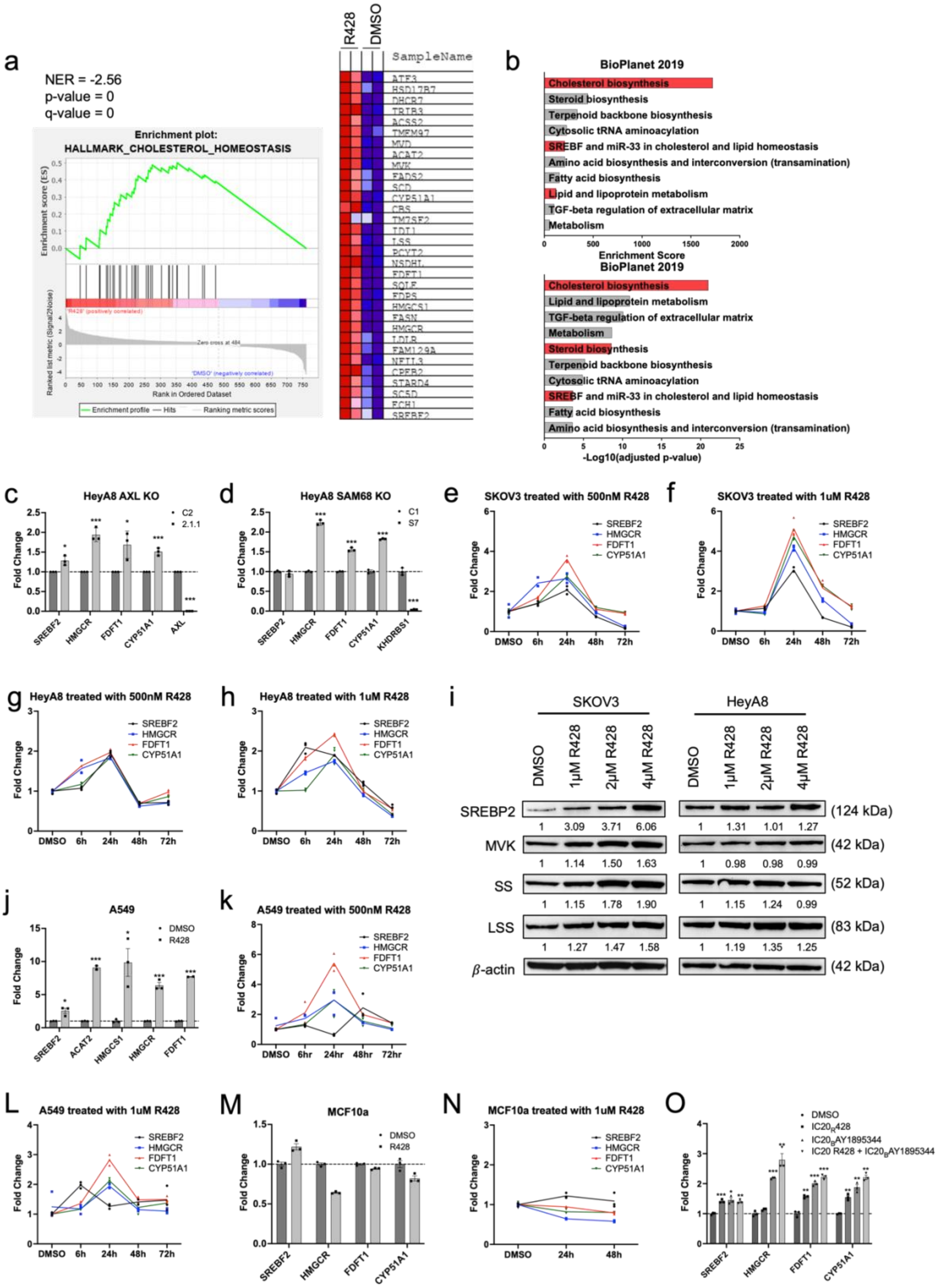
a
NER = -2.56
p-value = 0
q-value = 0
Enrichment plot: HALLMARK_CHOLESTEROL_HOMEOSTASIS
Enrichment score (ES)
Ranked list metric (Signal2Noise)
'R428' (positively correlated)
Zero cross at 484
'DMSO' (negatively correlated)
Rank in Ordered Dataset
Enrichment profile — Hits — Ranking metric scores
R428
DMSO
SampleName
ATF3
HSD17B7
DHCR7
TRIB3
ACSS2
TMEM97
MVD
ACAT2
MVK
FADS2
SCD
CYP51A1
CBS
TM7SF2
IDI1
LSS
PCYT2
NSDHL
FDFT1
SQLE
FDPS
HMGCS1
FASN
HMGCR
LDLR
FAM129A
NFIL3
CPEB2
STARD4
SC5D
ECH1
SREBF2
b
BioPlanet 2019
Cholesterol biosynthesis
Steroid biosynthesis
Terpenoid backbone biosynthesis
Cytosolic tRNA aminoacylation
SREBF and miR-33 in cholesterol and lipid homeostasis
Amino acid biosynthesis and interconversion (transamination)
Fatty acid biosynthesis
Lipid and lipoprotein metabolism
TGF-beta regulation of extracellular matrix
Metabolism
Enrichment Score
-Log10(adjusted p-value)
c
HeyA8 AXL KO
C2
2.1.1
Fold Change
SREBF2
HMGCR
FDFT1
CYP51A1
AXL
d
HeyA8 SAM68 KO
C1
S7
KHDRBS1
e
SKOV3 treated with 500nM R428
f
SKOV3 treated with 1uM R428
DMSO
6h
24h
48h
72h
g
HeyA8 treated with 500nM R428
h
HeyA8 treated with 1uM R428
i
SKOV3
HeyA8
DMSO
1μM R428
2μM R428
4μM R428
SREBP2
(124 kDa)
1 3.09 3.71 6.06
1 1.31 1.01 1.27
MVK
(42 kDa)
1 1.14 1.50 1.63
1 0.98 0.98 0.99
SS
(52 kDa)
1 1.15 1.78 1.90
1 1.15 1.24 0.99
LSS
(83 kDa)
1 1.27 1.47 1.58
1 1.19 1.35 1.25
β-actin
(42 kDa)
j
A549
DMSO
R428
SREBF2
ACAT2
HMGCS1
HMGCR
FDFT1
k
A549 treated with 500nM R428
6hr
24hr
48hr
72hr
L
A549 treated with 1uM R428
M
MCF10a
N
MCF10a treated with 1uM R428
O
DMSO
IC20$_R$428
IC20$_B$AY1895344
IC20 R428 + IC20$_B$AY1895344

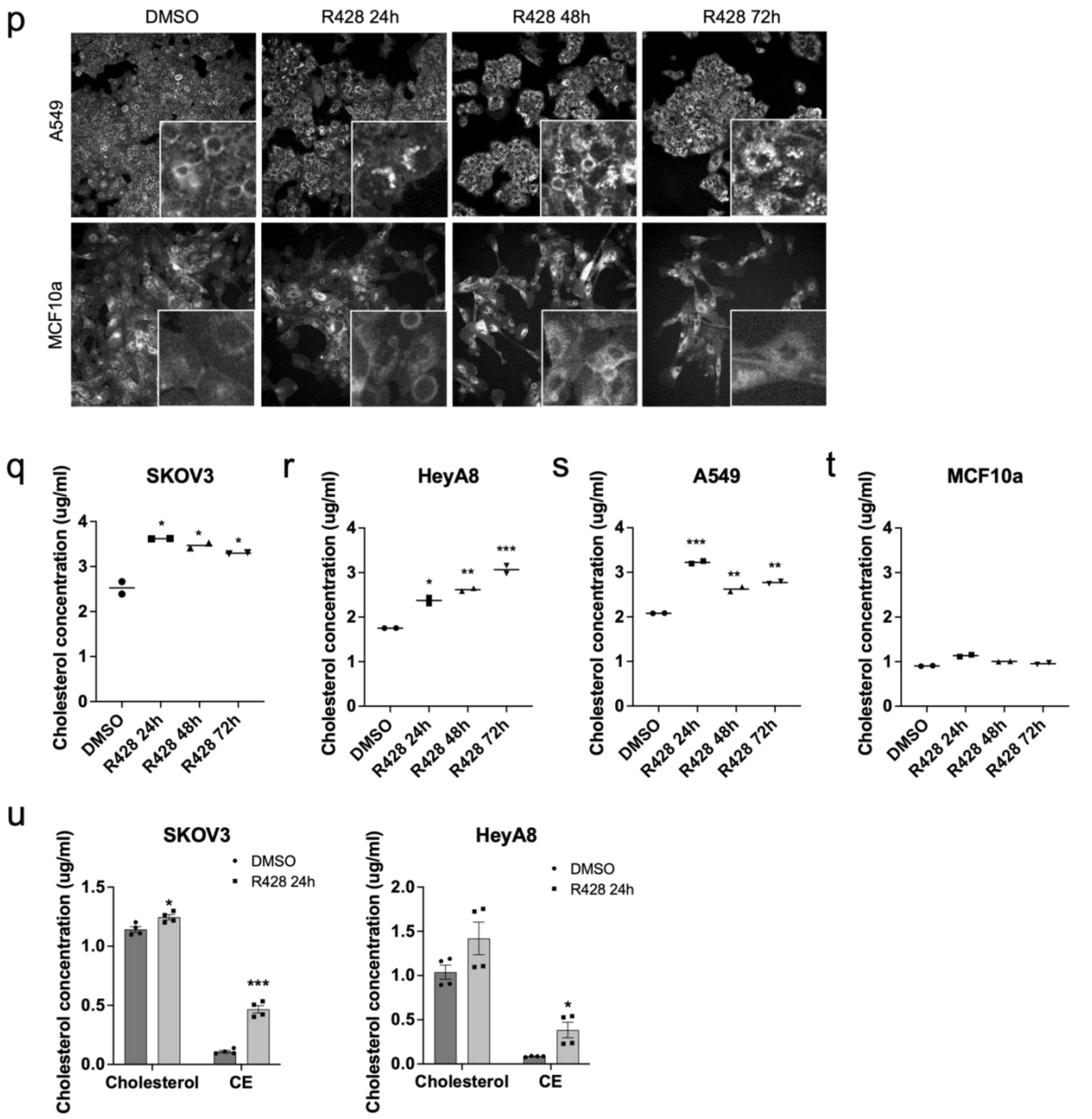

Supplementary figure 5: AXL KO, SAM68 KO and AXL inhibition increase cholesterol biosynthesis

(a) GSEA analysis showing positive enrichment of cholesterol homeostasis (b) Top 10 upregulated pathways upon AXL inhibition. Red indicates candidate pathways chosen for validation (c) Relative fold change of cholesterol biosynthesis genes in AXL KO and (d) SAM68 KO (e) Cholesterol biosynthesis genes expression peaked at 24 hrs upon AXL inhibition in a dose- and time-dependent manner in (e & f) SKOV3 and (g & h) HeyA8. (i) Immunoblot of dose-dependent upregulation of cholesterol biosynthesis protein expression levels in SKOV3 and HeyA8. Numbers below blots reflect protein band intensity normalised against DMSO (j) Relative fold change of cholesterol biosynthesis genes in A549 (k & l) Cholesterol biosynthesis genes expression peaked at 24 hrs upon AXL inhibition in a dose- and time-dependent manner in A549 (m) Relative fold change of cholesterol biosynthesis genes in

MCF10a (n) No time-dependent upregulation in expression of cholesterol biosynthesis genes in MCF10a (o) Upregulation of cholesterol biosynthesis genes upon combination treatment with R428 and BAY1895344 (p) Filipin III staining in A549 and MCF10a (q) Quantification of total cholesterol present in SKOV3 (r) HeyA8 (s) A549 (t) MCF10A after AXL inhibition (u) Quantification of free cholesterol and cholesteryl esters present in SKOV3 upon AXL inhibition; *p < 0.05, **p< 0.01, ***p < 0.001, determined by two-tailed t-test with Welch's correction

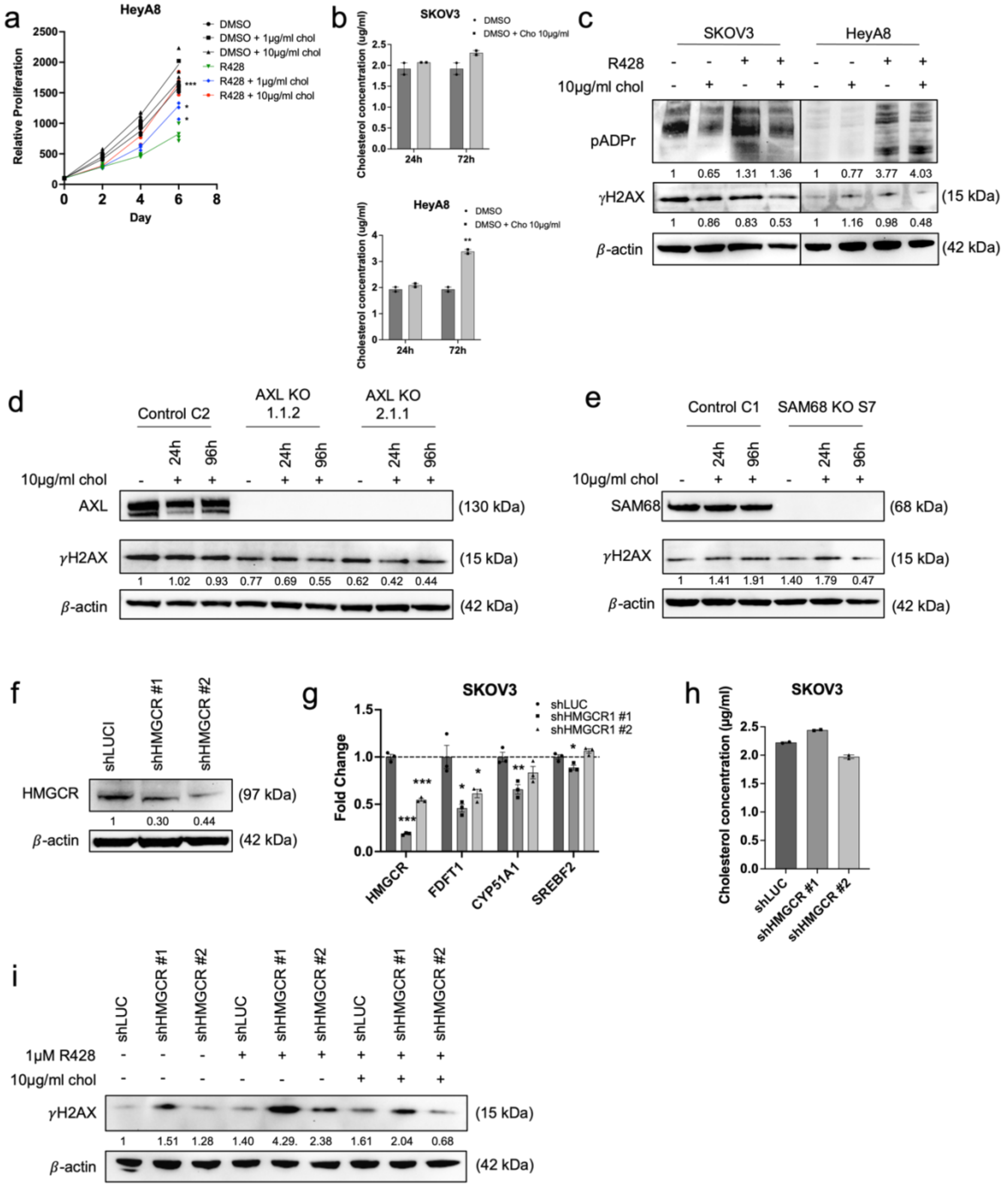

Supplementary figure 6: Increased cholesterol mitigates DNA damage induced by AXL inhibition

(a) Proliferation curve of HeyA8 treated with R428 supplemented with cholesterol; *$p < 0.05$, **$p < 0.01$, ***$p < 0.001$, determined by two-tailed t-test with statistical significance with Welch's correction (b) Quantification of total cholesterol in SKOV3 and HeyA8 supplemented with cholesterol (c) Immunoblot of SKOV3 and HeyA8 treated with R428 for 4 days, with and without supplementation of 10µg/ml cholesterol (d) Immunoblot of HeyA8 AXL KO cell lines and (e) SAM68 KO cell lines supplemented with and without supplementation of 10µg/ml cholesterol for 24 hrs and 96 hrs (f) HMGCR knockdown in SKOV3 (shHMGCR) (g) Relative expression levels of cholesterol biosynthesis genes in shHMGCR (h) Quantification of total cholesterol in shHMGCR cell lines; *$p < 0.05$, **$p < 0.01$, ***$p < 0.001$, determined by two-tailed t-test with Welch's correction (i) Immunoblot of shHMGCR cell lines treated with R428 with or without supplementation of 10 µg/ml cholesterol for 3 days. Numbers below blots reflect protein band intensity normalised against respective controls; Chol: cholesterol

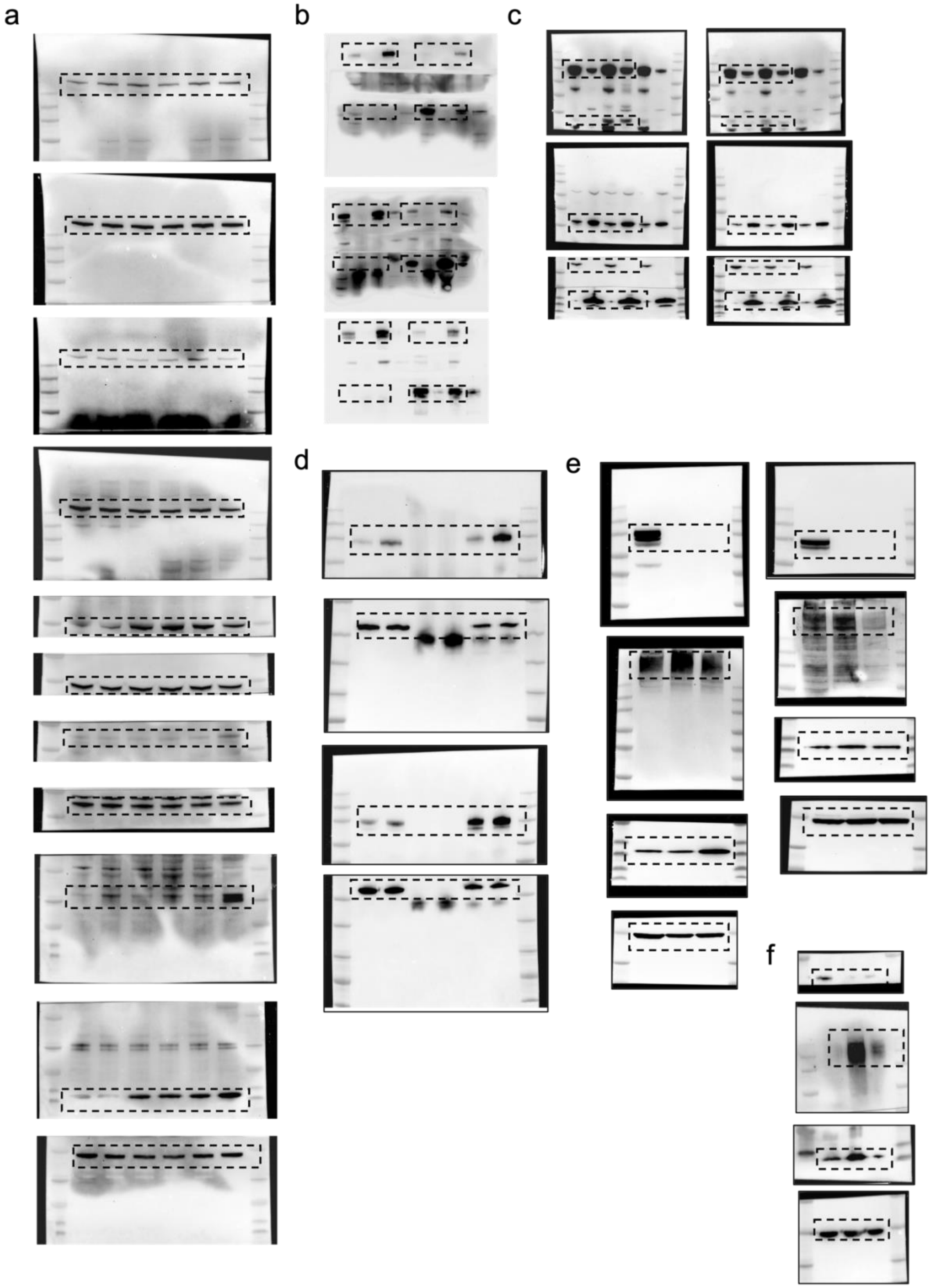

Supplementary figure 7: Full western blots

(a) Original full western blots shown in Figure 2e (b) Original full western blots shown in Figure 5b (c) Original full western blots shown in Figure 5c (d) Original full western blots

shown in Figure 5d (e) Original full western blots shown in Figure 6a (f) Original full western blots shown in Figure 6b

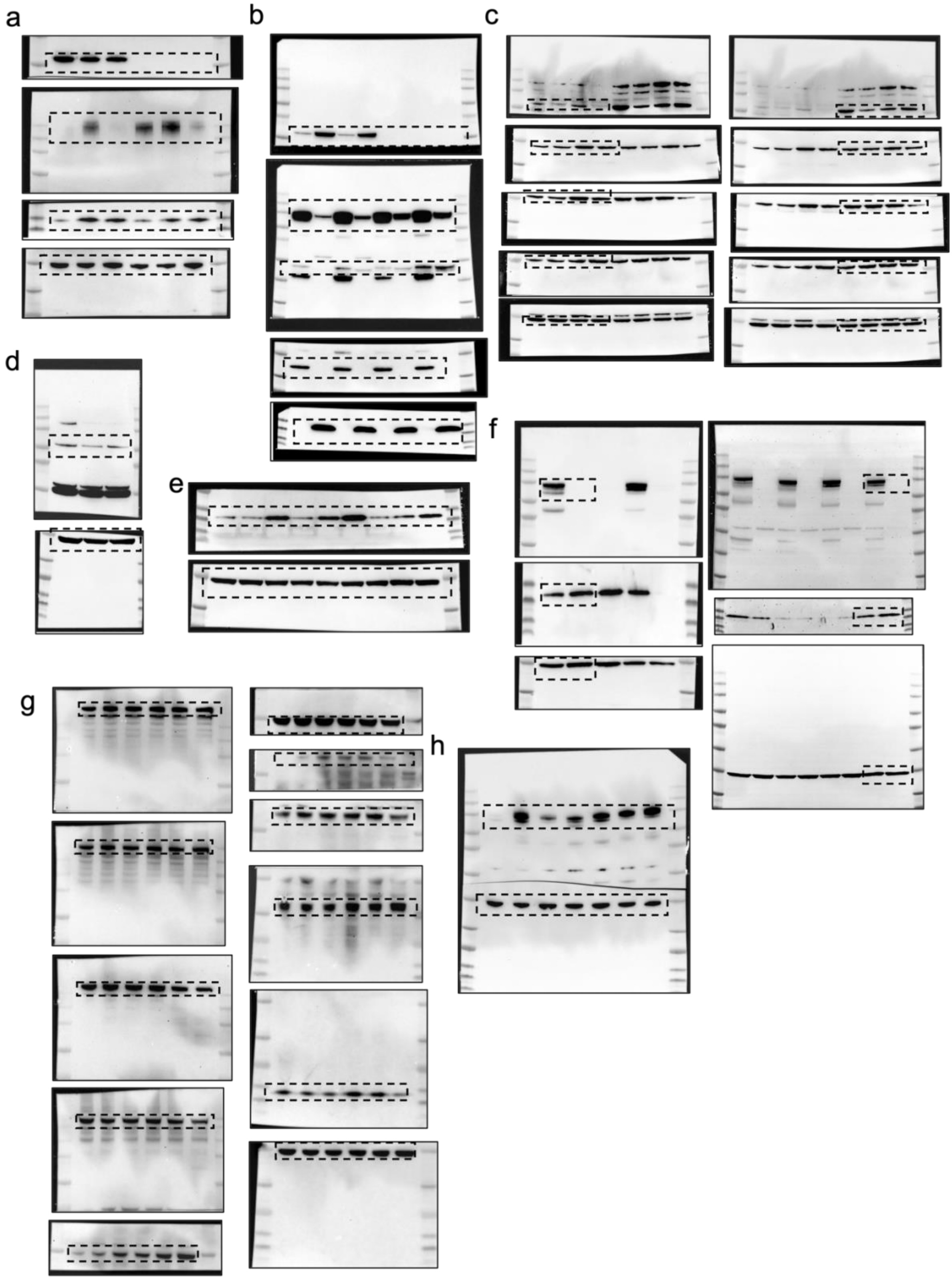

Supplementary figure 8: Full western blots

(a) Original full western blots shown in Figure 6c (b) Original full western blots shown in Figure 6d (c) Original full western blots shown in Figure 7f (d) Original full western blots shown in Figure 8b (e) Original full western blots shown in Figure 8e (f) Original full western blots shown in Supp figure 2a (g) Original full western blots shown in Supp figure 2h (h) Original full western blots shown in Supp figure 3a

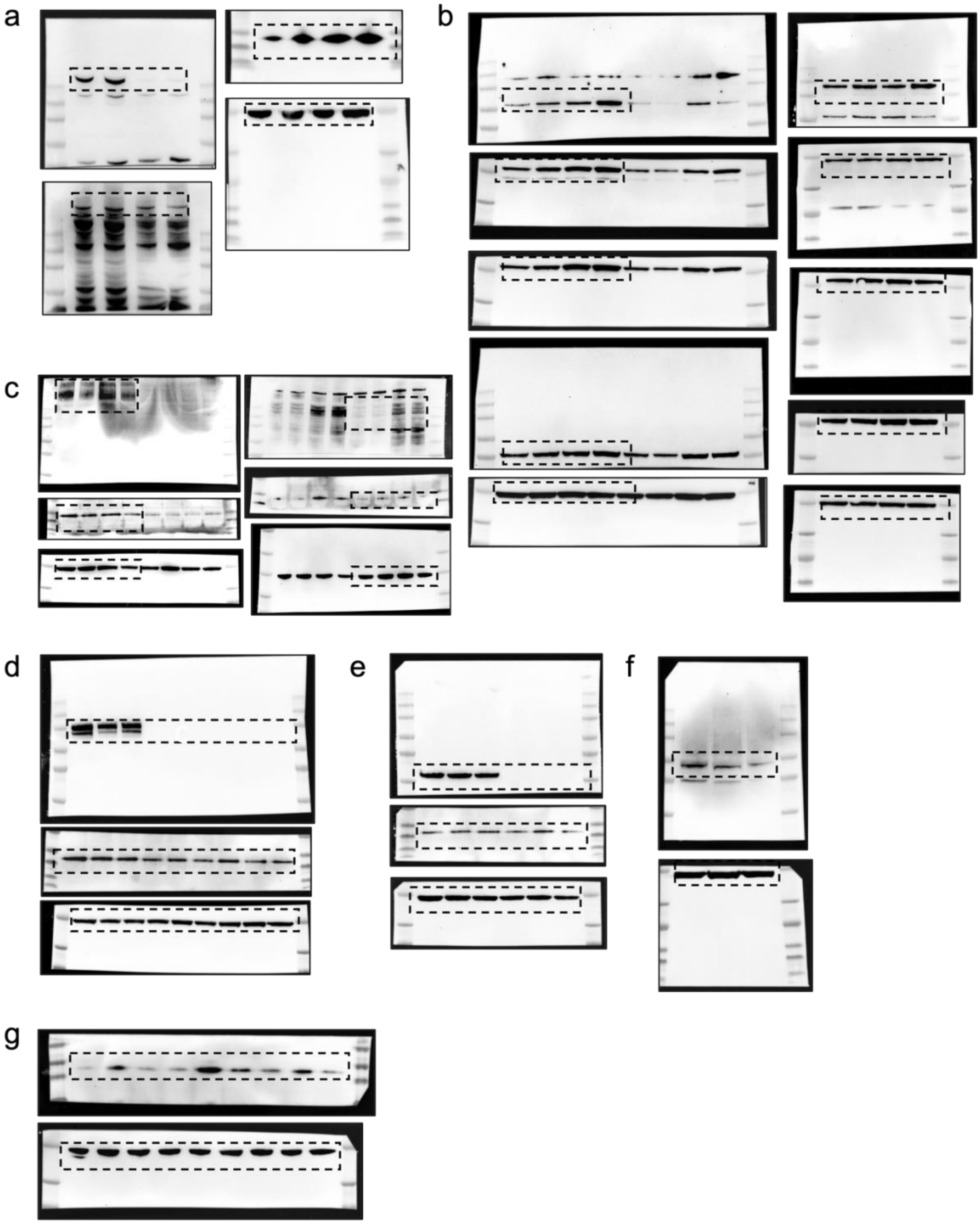

Supplementary figure 9: Full western blots

(a) Original full western blots shown in Supp figure 3n (b) Original full western blots shown in Supp figure 6i (c) Original full western blots shown in Supp figure 7c (d) Original full

western blots shown in Supp figure 7d (e) Original full western blots shown in Supp figure 7e (f) Original full western blots shown in Supp figure 7f (g) Original full western blots shown in Supp figure 7i

Supplementary Tables

Supplementary Table 1: List of shRNA sequences

Target Gene	shRNA ID	Target sequences
Luciferase	shLUC	CGCTGAGTACTTCGAAATGTC
AXL	shAXL#40	CGAAATCCTCTATGTCAACAT
SREBF2	shSREBF2#1	CCTGAGTTTCTCTCTCCTGAA
SREBF2	shSREBF2#2	GCAACAACAGACGGTAATGAT
HMGCR	shHMGCR#1	ATCAACTTGTGTACTGATAAA
HMGCR	shHMGCR#2	CAAGTTATTACCCTAAGTTTA

Supplementary Table 2: List of CRISPR knockout sgRNA sequences

Gene	sgRNA ID	sgRNA sequence
AXL Exon 1	1.1.2	GCGTGGCGGTGCCCCAGGAT
AXL Exon 2	2.1.22 / 2.1.1	GCTGAAGAAAGTCCCTTCGT
SAM68 Exon 5	S7	TGAACCCTCTCGTGGACGTG
SAM68 Exon 6	S8	TGGTGTTGGACCACCTCGGG

Supplementary Table 3: Sequence of primers used for qPCR

Gene	Forward Primer	Reverse Primer
GAPDH	ACAACTTTGGTATCGTGGAAGG	GCCATCACGCCACAGTTTC
AXL	GTGGGCAACCCAGGGAATATC	GTACTGTCCCGTGTCGGAAAG
SERBF1	ACAGTGACTTCCCTGGCCTAT	GCATGGACGGGTACATCTTCA A
SERBF2	CCTGGGAGACATCGACGAGAT	TGAATGACCGTTGCACTGAAG
ACAT2	GCGGACCATCATAGGTTCCTT	ACTGGCTTGTCTAACAGGATTC T
HMGCS1	CTCTTGGGATGGACGGTATGC	GCTCCAACTCCACCTGTAGG
HMGCR	TGATTGACCTTTCCAGAGCAAG	CTAAAATTGCCATTCCACGAGC
FDFT1	CCACCCCGAAGAGTTCTACAA	TGCGACTGGTCTGATTGAGATA

FASN	ACAGCGGGGAATGGGTACT	GACTGGTACAACGAGCGGAT
CYP51A1	GAAACGCAGACAGTCTCAAGA	ACGCCCATCCTTGTATGTAGC
KHDRBS1	TTGGTACGTGGTACACCAGTA	AGGCAAAGGTATCCTCTGGATG
