# Supplementary material for: The effect of inhibition of receptor tyrosine kinase AXL on DNA damage response in ovarian cancer
Source: Commun Biol. 2023 Jun 22;6:660. doi: 10.1038/s42003-023-05045-0 (PMC10287694; doi:10.1038/s42003-023-05045-0)
Supplement: Supplementary file 3 — Description of Additional Supplementary Files [file 42003_2023_5045_MOESM3_ESM.pdf]

# Description of Additional Supplementary Files

**File name:** Supplementary Data
**Description:** All source data underlying the graphs and charts presented in the main figures.
