# Supplementary material for: The effect of inhibition of receptor tyrosine kinase AXL on DNA damage response in ovarian cancer
Source: Commun Biol. 2023 Jun 22;6:660. doi: 10.1038/s42003-023-05045-0 (PMC10287694; doi:10.1038/s42003-023-05045-0)
Supplement: Supplementary file 5 — Reporting Summary [file 42003_2023_5045_MOESM5_ESM.pdf]

nature portfolio

Corresponding author(s): COMMSBIO-22-2000

Last updated by author(s): Jun 9, 2023

Reporting Summary

Nature Portfolio wishes to improve the reproducibility of the work that we publish. This form provides structure for consistency and transparency in reporting. For further information on Nature Portfolio policies, see our Editorial Policies and the Editorial Policy Checklist.

Statistics

For all statistical analyses, confirm that the following items are present in the figure legend, table legend, main text, or Methods section.

n/a	Confirmed	
☐	☒	The exact sample size (*n*) for each experimental group/condition, given as a discrete number and unit of measurement
☐	☒	A statement on whether measurements were taken from distinct samples or whether the same sample was measured repeatedly
☐	☒	The statistical test(s) used AND whether they are one- or two-sided<br>*Only common tests should be described solely by name; describe more complex techniques in the Methods section.*
☐	☒	A description of all covariates tested
☐	☒	A description of any assumptions or corrections, such as tests of normality and adjustment for multiple comparisons
☐	☒	A full description of the statistical parameters including central tendency (e.g. means) or other basic estimates (e.g. regression coefficient) AND variation (e.g. standard deviation) or associated estimates of uncertainty (e.g. confidence intervals)
☐	☒	For null hypothesis testing, the test statistic (e.g. *F*, *t*, *r*) with confidence intervals, effect sizes, degrees of freedom and *P* value noted<br>*Give P values as exact values whenever suitable.*
☒	☐	For Bayesian analysis, information on the choice of priors and Markov chain Monte Carlo settings
☒	☐	For hierarchical and complex designs, identification of the appropriate level for tests and full reporting of outcomes
☒	☐	Estimates of effect sizes (e.g. Cohen's *d*, Pearson's *r*), indicating how they were calculated

Our web collection on statistics for biologists contains articles on many of the points above.

Software and code

Policy information about availability of computer code

Data collection	NA
Data analysis	GraphPad Prism software was used for bar/line graphs output and statistical analysis. FlowJo was used for flwow data analysis. The RNA-Seq data were analyzed and visualized by R software.

For manuscripts utilizing custom algorithms or software that are central to the research but not yet described in published literature, software must be made available to editors and reviewers. We strongly encourage code deposition in a community repository (e.g. GitHub). See the Nature Portfolio guidelines for submitting code & software for further information.

Data

Policy information about availability of data

All manuscripts must include a data availability statement. This statement should provide the following information, where applicable:

- Accession codes, unique identifiers, or web links for publicly available datasets
- A description of any restrictions on data availability
- For clinical datasets or third party data, please ensure that the statement adheres to our policy

Data available on request from the authors

Human research participants

Policy information about studies involving human research participants and Sex and Gender in Research.

Reporting on sex and gender	NA
Population characteristics	NA
Recruitment	NA
Ethics oversight	NA

Note that full information on the approval of the study protocol must also be provided in the manuscript.

Field-specific reporting

Please select the one below that is the best fit for your research. If you are not sure, read the appropriate sections before making your selection.

☒ Life sciences ☐ Behavioural & social sciences ☐ Ecological, evolutionary & environmental sciences

For a reference copy of the document with all sections, see nature.com/documents/nr-reporting-summary-flat.pdf

Life sciences study design

All studies must disclose on these points even when the disclosure is negative.

Sample size	Sample size was determined based on p-value < 0.05
Data exclusions	No data were excluded
Replication	Minimum two biological replicates with three technical replicates. Replications were successful
Randomization	Samples were treated/collected at different occassions
Blinding	Blinding was not relevant to study

Reporting for specific materials, systems and methods

We require information from authors about some types of materials, experimental systems and methods used in many studies. Here, indicate whether each material, system or method listed is relevant to your study. If you are not sure if a list item applies to your research, read the appropriate section before selecting a response.

Materials & experimental systems

n/a	Involved in the study	
☐	☒	Antibodies
☐	☒	Eukaryotic cell lines
☒	☐	Palaeontology and archaeology
☐	☒	Animals and other organisms
☒	☐	Clinical data
☒	☐	Dual use research of concern

Methods

n/a	Involved in the study	
☒	☐	ChIP-seq
☐	☒	Flow cytometry
☒	☐	MRI-based neuroimaging

Antibodies

Antibodies used	Primary antibodies used were anti-AXL (CS8661; C44E1), anti-ATM (92356S), anti-pATM (13050S), anti-ATR (13934S), anti-pATR (58014S), anti CHK1 (2360S), anti-pCHK1 (2348S), anti-CHK2 (6334S) and anti-pCHK2 (2197S) from Cell signalling; anti-SAM68 (07-415) and anti-γH2AX (3BW301) from Millipore; antiRPA2 [p Ser33] (NB100-544) from Novus Biologicals; anti-SAM68 (ab76471), anti-SREBP2 (30682), anti-HMGCR (242315) from Abcam; anti-squalene synthase (sc-271602), anti-MVK (sc-390669), anti-OSC (sc-514507), anti-pADPr (sc-56198), anti-GAPDH (sc-47724), anti-ß-actin (sc-47778) from Santa Cruz. Secondary antibodies used were HRP-conjugated goat anti-mouse and goat anti-rabbit from Cell signalling

Validation	Based on manufacturer's website and antibody profiles in Online databases

Eukaryotic cell lines

Policy information about cell lines and Sex and Gender in Research

Cell line source(s)	ATCC, ECACC, Kyoto University
Authentication	Cell lines were identified by STR and phenotype markers
Mycoplasma contamination	All cell lines tested negative for mycoplasma
Commonly misidentified lines (See ICLAC register)	NA

Animals and other research organisms

Policy information about studies involving animals; ARRIVE guidelines recommended for reporting animal research, and Sex and Gender in Research

Laboratory animals	Chicken egg CAM
Wild animals	Study did not involve wild animals
Reporting on sex	NA
Field-collected samples	Study did not involve field-collected samples
Ethics oversight	National Taiwan University

Note that full information on the approval of the study protocol must also be provided in the manuscript.

Flow Cytometry

Plots

Confirm that:

☒ The axis labels state the marker and fluorochrome used (e.g. CD4-FITC).

☒ The axis scales are clearly visible. Include numbers along axes only for bottom left plot of group (a 'group' is an analysis of identical markers).

☒ All plots are contour plots with outliers or pseudocolor plots.

☒ A numerical value for number of cells or percentage (with statistics) is provided.

Methodology

Sample preparation	Cells were treated accordingly and EdU labelling was carried out using Click-iT™ EdU Alexa Fluor™ 647 flow cytometry assay kit as per written in manufacturer's protocol
Instrument	LSRII Cell Analyzer
Software	FlowJo v10
Cell population abundance	10,000 events were recorded
Gating strategy	Cells were preliminary gated based on FCS/SSC of cell lines and and boundaries were set based on positive and negative staining populations

☐ Tick this box to confirm that a figure exemplifying the gating strategy is provided in the Supplementary Information.
